# A Multiphase CT-Based Integrated Deep Learning Framework for Rectal Cancer Detection, Segmentation, and Staging: Performance Comparison with Radiologist Assessment

**DOI:** 10.3390/jimaging12020076

**Published:** 2026-02-10

**Authors:** Tzu-Hsueh Tsai, Jia-Hui Lin, Yen-Te Liu, Jhing-Fa Wang, Chien-Hung Lee, Chiao-Yun Chen

**Affiliations:** 1 Graduate Institute of Clinical Medicine, College of Medicine, Kaohsiung Medical University, Kaohsiung City 807, Taiwan; u106801005@kmu.edu.tw; 2 Novatek Microelectronics Corp., Hsinchu 300, Taiwan; 3 Kaohsiung Medical University Hospital, Kaohsiung City 807, Taiwan; 4 Department of Electrical Engineering, National Cheng Kung University, Tainan 701, Taiwan; 5 Department of Public Health, College of Health Sciences, Kaohsiung Medical University, Kaohsiung City 807, Taiwan; 6 Research Center for Precision Environmental Medicine, Kaohsiung Medical University, Kaohsiung City 807, Taiwan; 7 Department of Medical Imaging, Kaohsiung Medical University Hospital, Kaohsiung City 807, Taiwan; 8 School of Post-Baccalaureate Medicine, College of Medicine, Kaohsiung Medical University, Kaohsiung City 807, Taiwan; 9 Drug Development and Value Creation Research Center, Kaohsiung Medical University, Kaohsiung City 807, Taiwan

**Keywords:** artificial intelligence, deep learning, convolutional neural networks, computed tomography, rectal cancer, image segmentation, cancer staging

## Abstract

Accurate staging of rectal cancer is crucial for treatment planning; however, computed tomography (CT) interpretation remains challenging and highly dependent on radiologist expertise. This study aimed to develop and evaluate an AI-assisted system for rectal cancer detection and staging using CT images. The proposed framework integrates three components—a convolutional neural network (RCD-CNN) for lesion detection, a U-Net model for rectal contour delineation and tumor localization, and a 3D convolutional network (RCS-3DCNN) for staging prediction. CT scans from 223 rectal cancer patients at Kaohsiung Medical University Chung-Ho Memorial Hospital were retrospectively analyzed, including both non-contrast and contrast-enhanced studies. RCD-CNN achieved an accuracy of 0.976, recall of 0.975, and precision of 0.976. U-Net yielded Dice scores of 0.897 (rectal contours) and 0.856 (tumor localization). Radiologist-based clinical staging had 82.6% concordance with pathology, while AI-based staging achieved 80.4%. McNemar’s test showed no significant difference between the AI and radiologist staging results (*p* = 1.0). The proposed AI-assisted system achieved staging accuracy comparable to that of radiologists and demonstrated feasibility as a decision-support tool in rectal cancer management. This study introduces a novel three-stage, dual-phase CT-based AI framework that integrates lesion detection, segmentation, and staging within a unified workflow.

## 1. Introduction

Colorectal cancer is the third most common malignancy globally, accounting for approximately 10% of all cancer cases and ranking as the second leading cause of cancer-related mortality [1]. Accurate staging of rectal cancer is essential for treatment planning and prognostication. Precise staging guides clinical decisions regarding the timing of surgery, the necessity of neoadjuvant therapy, and appropriate follow-up strategies, thereby holding substantial clinical value.

Imaging plays a central role in the diagnosis and staging of rectal cancer. Magnetic resonance imaging (MRI) is widely regarded as the gold standard for local staging due to its superior soft-tissue contrast [2,3,4,5]. However, in routine clinical practice, MRI has several limitations, including high cost, longer acquisition and waiting times, and limited accessibility in many healthcare settings. In contrast, computed tomography (CT) is more accessible and faster to perform and, when combined with rectal insufflation [6], can improve bowel distension and lesion visualization—making it a practical alternative in real-world scenarios. Meta-analyses have shown that while MRI generally outperforms CT in local staging accuracy [3,4], optimized CT protocols can achieve clinically acceptable diagnostic performance [7,8], particularly when interpreted by experienced radiologists. The Society of Abdominal Radiology recognizes CT as an important staging tool in settings where MRI is unavailable or contraindicated [7]. However, CT interpretation remains highly dependent on radiologist expertise, and less experienced readers may demonstrate substantial variability in staging accuracy.

Recent advances in artificial intelligence (AI) and deep learning have significantly impacted medical imaging, with successful applications in disease classification, tumor detection, and organ segmentation [9,10,11]. Convolutional neural networks (CNNs), including their three-dimensional extensions (3D CNNs), have shown promise in enhancing CT-based diagnostic accuracy [12,13]. In particular, U-Net and its variants have become state-of-the-art models for medical image segmentation, enabling precise delineation of anatomical structures and tumor boundaries [14,15,16]. Several studies have explored deep learning approaches for tumor detection and segmentation in colorectal and rectal cancer [17,18,19], with most efforts focused on MRI or PET. Recent work has also investigated radiomics and deep learning for response prediction and staging in rectal cancer [9]. However, comprehensive evaluations of AI-based staging using CT imaging remain limited, especially those directly comparing AI models with radiologist performance using pathology as the reference standard.

In this study, we present an integrated AI framework specifically tailored to a dual-phase, dual-position CT protocol. Unlike standard CT approaches, this study leverages the complementary information of prone non-contrast and supine contrast-enhanced acquisitions to assess the feasibility of automated rectal cancer evaluation. The proposed system consists of three components: (1) a Rectal Cancer Detection Convolutional Neural Network (RCD-CNN) for automatic lesion detection, (2) a U-Net-based model for rectal contour delineation and tumor localization, and (3) a 3D CNN (RCS-3DCNN) for staging prediction. Furthermore, AI-based staging results were directly compared with clinical assessments by radiologists, using pathological diagnosis as the reference standard, to evaluate the feasibility of using AI as a decision-support tool in rectal cancer management.

## 2. Materials and Methods

### 2.1. Study Design and Patient Population

This retrospective study was approved by the Institutional Review Board of Kaohsiung Medical University Chung-Ho Memorial Hospital (KMUHIRB-20200444), with the requirement for informed consent waived owing to the retrospective nature of the investigation.

Inclusion criteria were as follows:

(1) newly diagnosed rectal adenocarcinoma confirmed by colonoscopy and biopsy between January 2013 and November 2019;
(2) pathological T stage of T1–T3;
(3) availability of complete pre- and post-contrast CT examinations; and
(4) surgical resection performed within 30 days after CT without any intervening neoadjuvant therapy.

Exclusion criteria were as follows:

(1) prior neoadjuvant chemotherapy or radiotherapy;
(2) recurrent rectal cancer;
(3) severe image artifacts that compromised interpretation;
(4) incomplete pathological reports; and
(5) T4 disease at presentation.

Patients with T4 disease were excluded for two reasons. First, the distinction between T2 and T3 presents a greater diagnostic challenge and determines the indication for neoadjuvant therapy. Second, and methodologically more critical, T4 patients typically undergo neoadjuvant chemoradiotherapy prior to surgery. This treatment alters the tumor stage (downstaging), meaning the post-operative pathological stage (ypT) would not serve as an accurate reference standard for the pre-treatment CT images. Excluding these cases ensured a direct and accurate correlation between imaging features and pathological ground truth.

A total of 223 patients met these criteria and were included in the study. Each patient underwent dual-phase, dual-position rectal CT examinations, consisting of (1) a non-contrast prone acquisition and (2) a contrast-enhanced supine acquisition after rectal insufflation. In the prone position, the rectum was elevated, allowing insufflated air to accumulate under gravity, thereby facilitating luminal distension and improving delineation of the rectal wall. Conversely, the contrast-enhanced scan was performed in the supine position, where tumor enhancement could be better visualized, and gravitational shifting of fecal material helped distinguish tumor tissue from residual stool. This dual-position protocol was designed to be complementary, maximizing rectal wall visualization and overall tumor conspicuity.

**Figure 1:**
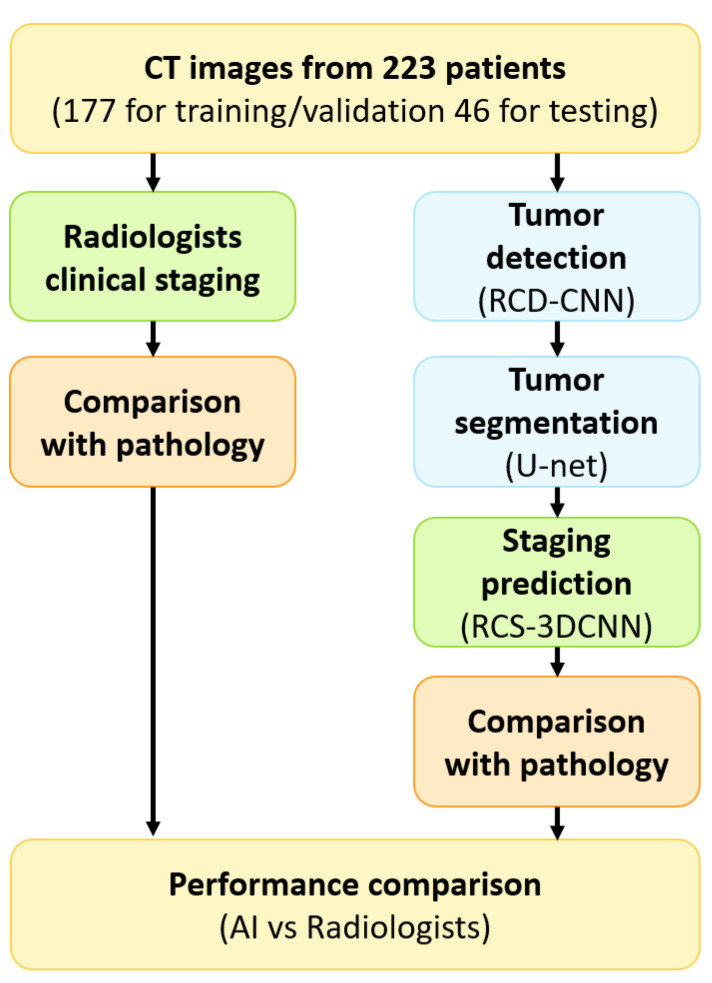
Overall workflow of the study, showing patient allocation, AI pipeline (RCD-CNN for detection, U-Net for segmentation, RCS-3DCNN for staging), and comparison with pathology and radiologists. Colors are used for visual workflow illustration only: blue indicates AI preprocessing (e.g., segmentation), green indicates AI or radiologist assessment, and orange indicates pathology comparison; yellow boxes are for visual emphasis only.

Pathology was used as the reference standard. The study was designed as a binary classification task (T1/T2 vs. T3) a priori. This grouping aligns with clinical management guidelines where the distinction between early-stage (T1/T2) and locally advanced (T3) disease dictates the decision for neoadjuvant therapy. Furthermore, this binary formulation maximized statistical power given the available sample size. To prevent information leakage, dataset partitioning was performed at the patient level, with patients randomly assigned to training, validation, and testing sets in approximately a 7:1:2 ratio (*n* = 155, 22, and 46, respectively). An independent held-out test subset (*n* = 46) with both clinical staging and pathology results was used for direct comparison between AI staging and radiologists’ clinical staging. An overview of the study workflow, including patient allocation, AI pipeline, and comparison with pathology and radiologists, is illustrated in Figure 1.

### 2.2. CT Acquisition Protocol

All CT examinations were performed using a 128-row dual-source, dual-energy multidetector CT scanner (SOMATOM Definition Flash; Siemens Medical Solutions, Forchheim, Germany). Acquisition parameters included a tube voltage of 120 kVp and a quality reference mAs of 250 with automatic exposure control (CAREDose 4D; Siemens Healthcare, Erlangen, Germany).

Each patient was positioned in the left lateral decubitus position, and a 12-F balloon-tipped rectal tube was inserted. Approximately 1200 mL of room air was gently insufflated to achieve adequate colonic distension and facilitate tumor visualization [6]. A scout image was obtained to confirm satisfactory distension, defined as clear visualization of all colonic segments. Additional insufflation was performed when necessary. No insufflation-related complications occurred.

For the non-contrast scan, patients were repositioned prone to allow air accumulation in the rectum. The scan range extended from the third lumbar vertebra (L3) to the perineum. Subsequently, 100 mL of nonionic iodinated contrast material (Ultravist 300; Bayer, Berlin, Germany) was administered intravenously at 3 mL/s using an automatic injector. Spiral CT was then acquired during the portal venous phase (70 s after injection), covering the region from the hepatic dome to the perineum.

### 2.3. Image Annotation and Preprocessing

Axial CT images were de-identified and annotated using 3D Slicer (version 4.11; https://www.slicer.org; accessed on 6 February 2026). Two board-certified abdominal radiologists (14 and 30 years of experience, respectively) independently delineated tumor regions with pathology reports as the reference standard. Discrepancies were resolved by consensus, and the pathology-informed masks were used as ground truth for training and evaluation. DICOM images were encoded as 12-bit data with pixel values ranging from 0 to 4096. Images were first resampled to 512 × 512 pixels, then normalized to the range [0, 255], and finally converted to PNG format. Hounsfield units were windowed to −300 to 200 to emphasize rectal wall and tumor boundaries.

### 2.4. Data Augmentation and ROI Processing

To enhance model generalization and reduce overfitting during training, data augmentation techniques were applied, including random rotation (±20°), width shift (±0.05), height shift (±0.05), shear (±0.05), zoom (±0.05), and horizontal flipping. No augmentation was applied during validation or testing. Because rectal tumors occupy a relatively small proportion of each slice, regions of interest (ROIs) were extracted by first performing rectal contour segmentation, followed by computing the maximum bounding rectangle to isolate tumor areas. For ROI sequence processing, the number of tumor slices per patient varied. To ensure a fixed input sequence length for the RCS-3DCNN: (1) during training, random duplication with augmentation was applied to sequences with fewer than 12 slices to reach the required length; (2) during validation and testing, to maintain consistency, deterministic padding (repeating slices) was used to reach 12 slices. Finally, 12 slices with contrast and 12 without contrast were combined, yielding a fixed total of 24 ROI slices per patient for all datasets. A fixed input length of 24 slices was selected to balance volumetric coverage with computational efficiency. Dataset inspection confirmed that 24 slices (covering approximately 12 cm of craniocaudal extent) fully captured all tumors. Deterministic padding (slice repetition) was used for smaller tumors to preserve spatial texture continuity for the 3D-CNN, avoiding the introduction of artificial zero-value artifacts.

### 2.5. Model Architectures

**Figure 2:**
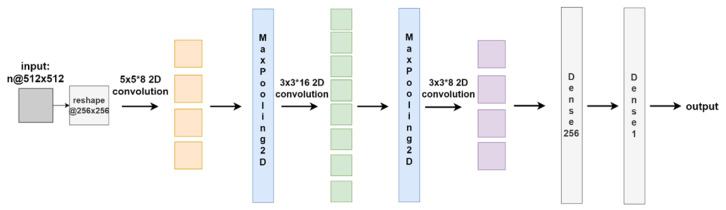
The Network Architecture of RCD-CNN (Input dimension: 256 × 256; Output: Binary tumor detection map). Arrows indicate processing flow direction. Colors are used for visual layer grouping only and do not represent categorical outputs.

**Figure 3:**
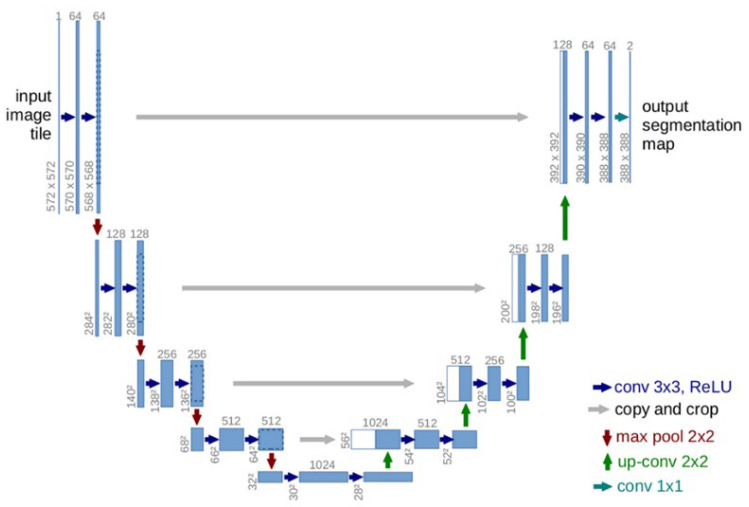
The Network Architecture of U-Net (Input dimension: 512 × 512; Output: Segmentation masks for rectal contour and tumor). Superscript “^2^” denotes squared spatial dimensions (e.g., 572^2^ = 572 × 572 pixels).

**Figure 4:**
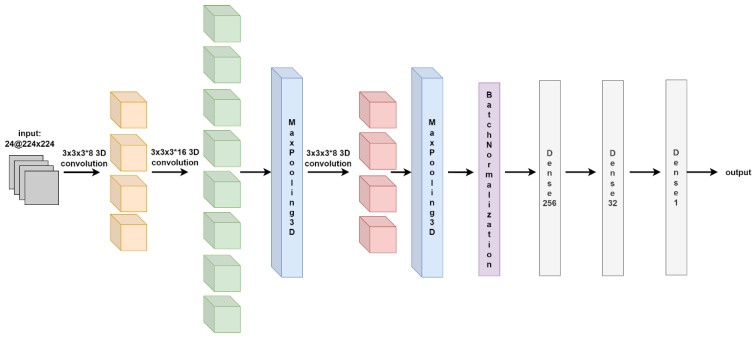
The Network Architecture of RCS-3DCNN (Input dimension: 24 × 224 × 224; Output: Binary classification probability). Arrows indicate processing flow direction. Colors are used for visual layer grouping only and do not represent categorical outputs.

(1) RCD-CNN: The RCD-CNN model was designed to detect tumor regions in axial CT slices. The architecture consisted of three convolution–pooling blocks with channel dimensions of 8, 16, and 8, and kernel sizes of 5 × 5, 3 × 3, and 3 × 3, respectively. A stride of 1 was used in all convolutional layers. Zero padding was not applied, as rectal tumors were consistently located in the central region of the image following rectal insufflation, making peripheral image content less informative. Max pooling layers were used for down-sampling. The convolutional blocks were followed by two fully connected layers with 256 and 1 neurons, respectively. The ReLU activation function was applied throughout the network. The overall network structure is shown in Figure 2.

(2) U-Net: The U-Net model was used for rectal contour extraction and tumor localization. The architecture consisted of two main parts: a contracting path (encoder) and an expansive path (decoder). The contracting path was responsible for feature extraction through a series of down-sampling operations achieved via convolutional layers and max pooling. It comprised four blocks, each consisting of two convolutional layers followed by a max pooling operation. With each down-sampling step, the feature map size was reduced by half, resulting in a final feature map size of 32 × 32. The expansive path aimed to reconstruct the segmentation mask from the compressed representation. It also consisted of four blocks that performed up-sampling operations through transpose convolution. Skip connections were used to concatenate feature maps from the contracting path with the corresponding up-sampled feature maps in the expansive path, preserving high-resolution information and enabling recovery of fine details. Finally, a 1 × 1 convolutional layer produced the output with two channels representing the foreground and background. The detailed architecture is illustrated in Figure 3.

(3) RCS-3DCNN: The RCS-3DCNN was developed for staging prediction, consisting of three 3D convolutional layers and two 3D max-pooling layers. The first layer had 8 channels with a filter size of 3 × 3 × 3, followed by the second layer with 16 channels and the same filter size. These two sets of 3D convolutions extracted spatial-temporal features, followed by a 3D max-pooling layer for down-sampling. The output was then connected to the third convolutional layer with 8 channels and a filter size of 3 × 3 × 3. Another 3D max-pooling layer was applied to further extract features. Batch normalization was incorporated after each convolutional layer to stabilize and normalize the learning process, contributing to improved training and generalization performance. The output from the final max-pooling layer was flattened and connected to three fully connected layers with 256, 32, and 1 neuron, respectively. These fully connected layers captured higher-level representations and produced a one-dimensional output for binary classification between T12 and T3. By employing 3D convolutions and max pooling, the RCS-3DCNN architecture could effectively capture spatial and temporal information from the dual-phase CT image sequences. The architecture of the proposed 3D network is shown in Figure 4.

### 2.6. Model Training and Implementation

Training was conducted on workstations with Intel i9-9900K CPU (Intel, Santa Clara, CA, USA), 64 GB RAM, and NVIDIA RTX 2080Ti GPU (NVIDIA, Santa Clara, CA, USA) under Linux/Windows. Batch sizes were set to 12 for RCD-CNN, 4 for U-Net, and 8 for RCS-3DCNN. Training was set to a maximum of 200 epochs.

For RCD-CNN, images were resized from 512 × 512 to 256 × 256 before training. The Adam optimizer was used with an initial learning rate of 0.001. A class weight ratio of 1:5 was applied to address the imbalance between tumor and non-tumor slices, and the loss function was weighted binary cross-entropy.

For U-Net, training was performed separately for rectal contour extraction (learning rate 3 × 10^−5^) and tumor localization (learning rate 1 × 10^−5^), both using the Adam optimizer. Dice coefficient loss was employed. ROI preprocessing was applied before training. Early stopping was applied if validation loss did not improve for 10 consecutive epochs, and the learning rate was adjusted using the ReduceLROnPlateau strategy (patience = 5).

For RCS-3DCNN, sequences of 24 ROI slices per patient were used as input. Before feeding into the network, the extracted ROIs were resized to 224 × 224 pixels to match the input dimensions of the RCS-3DCNN architecture. The Adam optimizer was applied with an initial learning rate of 0.01 and exponential decay. Binary cross-entropy was used as the loss function, with batch normalization to stabilize training. Early stopping and model checkpointing were employed to save the best-performing weights.

### 2.7. Evaluation Metrics and Statistical Analysis

For RCD-CNN, the accuracy, recall, and precision were calculated. For U-Net, the Dice similarity coefficient was reported. For RCS-3DCNN, the primary endpoint was distinguishing between T3 and T12 at the patient level using pathology as the reference. Sensitivity, specificity, and accuracy were reported with 95% confidence intervals (Wilson method). Binary classification metrics were specifically selected to facilitate a direct, head-to-head comparison with the categorical staging results provided by the radiologists, ensuring consistency in performance evaluation. Statistical significance was defined as *p* < 0.05. Agreement was assessed by Cohen’s kappa, and McNemar’s exact test (two-sided) was used to test paired proportions. For baseline characteristics, continuous variables were compared using independent *t*-test or Mann–Whitney U test as appropriate, and categorical variables were compared using chi-square or Fisher’s exact test.

### 2.8. Software and Availability

All preprocessing and annotation tasks were conducted using publicly available tools, including 3D Slicer (version 4.11; https://www.slicer.org; accessed on 6 February 2026). Source code is available upon reasonable request to the corresponding author. This study was not preregistered. Generative AI tools (ChatGPT, OpenAI, San Francisco, CA, USA) were only used for language editing and were not involved in study design, data acquisition, analysis, or interpretation.

## 3. Results

### 3.1. Patient Characteristics

A total of 223 patients with newly diagnosed rectal cancer were included, with a mean age of 63.0 ± 11.0 years; 134 (60%) were male. Pathological staging comprised 57 (25.6%) T1, 57 (25.6%) T2, and 109 (48.9%) T3 cases. The mean maximum tumor diameter was 3.9 ± 1.2 cm, and the median CEA level was 2.63 (IQR: 1.49–5.03) ng/mL. Among the entire cohort, 177 patients were assigned to the training and validation groups, and 46 patients to the independent test group. The test subset (mean age 63.7 ± 9.4 years; 65% male) showed a comparable distribution of pathological stages (30.4% T1, 28.3% T2, and 41.3% T3), indicating that it was representative of the overall cohort (Table 1). Although the CT–surgery interval was statistically shorter in the test cohort (*p* = 0.002), both intervals (3.5 and 6.6 days) are considered clinically acceptable short-term windows and are not expected to substantially impact the accuracy of T-stage assessment.

### 3.2. Lesion Detection

The proposed Rectal Cancer Detection CNN (RCD-CNN) demonstrated high performance in identifying rectal cancer lesions. On the independent test dataset, the model achieved an accuracy of 97.6%, recall of 97.5%, and precision of 97.6%, confirming its robust capability in distinguishing rectal tumor slices from normal tissue.

### 3.3. Segmentation Performance

For structural segmentation, U-Net models were evaluated separately for rectal contour extraction and tumor localization. The rectal contour model achieved a mean Dice similarity coefficient of 0.897, while the tumor localization model achieved a mean Dice coefficient of 0.856, indicating accurate delineation of both anatomical structures and tumor regions (Table 2).

### 3.4. Cancer Staging

Pathological diagnosis served as the reference standard for evaluating both radiologist-based clinical staging and AI-based staging. Clinical staging was concordant with pathology in 38 of 46 cases, yielding an accuracy of 82.6% (95% CI: 69.3–90.9%). AI staging was concordant in 37 of 46 cases, with an accuracy of 80.4% (95% CI: 66.8–89.3%). McNemar’s test indicated no significant difference between clinical and AI performance (*p* = 1.0). Agreement analysis demonstrated a Cohen’s kappa of 0.66 for clinical staging (substantial agreement) and 0.59 for AI staging (moderate agreement). These findings suggest that AI staging performance closely approaches the diagnostic level of radiologists (Table 3).

## 4. Discussion

This study developed and validated an integrated AI system for CT-based rectal cancer detection and staging. The framework demonstrated robust performance across all tasks, including lesion detection (97.6% accuracy), segmentation (Dice coefficient of 0.897 for rectal contours and 0.856 for tumors), and staging (80.4% accuracy). Notably, AI staging accuracy was comparable to that of experienced radiologists (82.6%, *p* = 1.0). Agreement analysis showed substantial concordance for radiologists (κ = 0.66) and moderate concordance for AI (κ = 0.59) when compared with pathology.

### 4.1. Technical Contributions and Comparison with Prior Work

The primary contribution of this study lies in developing an integrated three-stage pipeline that provides end-to-end automation—from raw CT images to staging predictions. Unlike previous studies that addressed isolated tasks [16,17,18,19], the proposed framework replicates the entire clinical workflow.

Our dual-phase CT protocol combines non-contrast prone and contrast-enhanced supine acquisitions with rectal insufflation [6], addressing CT’s intrinsic limitation of lower soft-tissue contrast compared with MRI. The prone acquisition facilitates gravitational air accumulation, improving luminal distension, whereas the supine contrast-enhanced acquisition enables better tumor enhancement and discrimination from fecal material.

The segmentation performance (Dice 0.897 for rectal contours, 0.856 for tumors) favorably compares with previous segmentation studies (typically 0.70–0.80) [16,17,18] and approaches the accuracy achieved by MRI-based systems [17,18,19]. The RCS-3DCNN architecture leverages volumetric information across 24 slices to capture three-dimensional tumor extent, enabling precise assessment of mural invasion depth—critical for differentiating T2 from T3 disease [13].

The staging accuracy of 80.4% is consistent with CT performance reported in systematic reviews (75–85%) [4], although it remains slightly lower than that of MRI-based staging (85–90%) [4,5]. The direct head-to-head comparison with radiologists using pathology as reference confirms the clinical feasibility of AI-assisted staging.

### 4.2. Clinical Rationale for CT-Based AI

While MRI remains the reference standard for rectal cancer staging [2,4,5,20], CT-based AI addresses several important clinical needs. CT is routinely performed in newly diagnosed colorectal cancer patients for initial staging, is widely available, and can be used when MRI is contraindicated (e.g., pacemakers, claustrophobia) or unavailable. In resource-limited settings, CT may represent the only cross-sectional imaging modality accessible [6,7].

CT-based AI can play complementary roles in: (1) triaging patients for urgent MRI, (2) providing initial staging while awaiting MRI, (3) serving as a primary staging tool when MRI is unavailable, (4) supporting less experienced radiologists, and (5) functioning as a quality-assurance tool to reduce inter-observer variability. These applications are consistent with AI-assisted workflows designed to augment, rather than replace, human expertise [9,10,21]. Furthermore, the proposed framework may have potential relevance for radiotherapy workflows, in which CT remains the standard modality for dose calculation and target volume definition. Although radiotherapy applications were not directly evaluated in this study, the tumor localization performance (Dice = 0.856) suggests that the generated masks could serve as an initial reference for gross tumor volume (GTV) delineation, with final contours determined by expert review.

### 4.3. Error Patterns and Human–AI Collaboration

**Figure 5:**
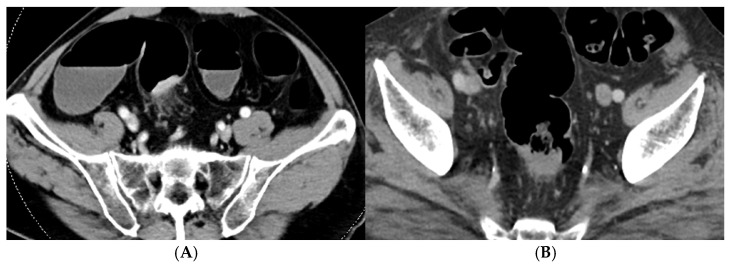
Representative misclassification examples illustrating the T2/T3 diagnostic challenge. (**A**) Radiologist over-staging: a T2 tumor was incorrectly classified as T3 due to dense perirectal stranding mimicking deep mural invasion. (**B**) AI under-staging: a T3 tumor was misclassified as T2, likely due to the subtle contrast difference at the tumor border which failed to trigger the staging threshold.

Error analysis showed that radiologists tended to overstage equivocal cases, whereas AI showed a more balanced distribution of over- and under-staging errors, suggesting distinct decision biases rather than complementary error patterns (Figure 5). Radiologists tended to overstage (7/8 errors, 87.5%, were T1–T2 misclassified as T3), likely reflecting clinical caution when mural invasion was equivocal. In contrast, AI errors were balanced (4/9 over-staging, 5/9 under-staging), suggesting consistent decision boundaries without clinical bias.

Importantly, when both radiologist and AI predictions agreed (*n* = 35, 76.1% of cases), the concordant prediction was correct in 94.3% (33/35 against pathology). This finding suggests that concordant predictions may indicate high-confidence cases, whereas discordant cases should prompt further review or MRI confirmation. Such hybrid human–AI workflows have shown promise in other imaging domains [9,21] and merit prospective validation.

The T2–T3 boundary remains a well-recognized diagnostic challenge even on MRI [22], as distinguishing subserosal invasion (T3) from muscularis propria involvement (T2) requires subtle assessment of tissue planes. Representative misclassification examples (Figure 5) illustrate this diagnostic difficulty. Although our AI model captured relevant features, it could not fully overcome this inherent limitation.

### 4.4. Limitations and Future Directions

This study has several limitations. First, the single-center retrospective design with a modest sample size (*n* = 223) limits generalizability. We acknowledge that the current results represent a proof-of-concept feasibility study. Future work utilizing multi-center data is essential to validate robustness across different scanners and protocols. Additionally, while rigorous data augmentation was employed to mitigate overfitting, k-fold cross-validation was not performed in this study to prioritize the use of a strictly held-out test set for the head-to-head comparison with radiologists. Furthermore, we employed lightweight custom model architectures rather than deep pre-trained networks (e.g., ResNet or EfficientNet) to prevent overfitting given the dataset size. Future studies with larger cohorts will explore transfer learning and more complex backbones to potentially enhance feature extraction capabilities. In this study, our primary contribution lies in the clinically motivated dual-phase CT workflow rather than architectural novelty; therefore, a standard U-Net was adopted as a strong and well-validated baseline. External validation on multi-center cohorts with heterogeneous acquisition protocols is essential prior to clinical implementation. Second, the disease spectrum was restricted to T1–T3 stages, excluding T4, post-neoadjuvant, and mucinous histologic subtypes, thereby limiting applicability to broader real-world scenarios.

Third, while AI staging accuracy approximated radiologist performance (80.4% vs. 82.6%), it did not exceed it. The clinical value of AI lies in consistent decision support—offering reproducible assessment, reducing inter-observer variability, flagging discordant cases, and operating continuously without fatigue. These advantages require demonstration through prospective workflow studies. Fourth, this study did not directly compare CT-based AI with MRI-based staging or explore multi-modal fusion approaches, which may further enhance performance. Fifth, practical deployment metrics such as inference time, PACS integration, workflow efficiency, and user acceptance were not formally evaluated.

Future research should prioritize (1) multi-center external validation across diverse CT protocols, (2) prospective reader studies comparing radiologist performance with and without AI assistance, (3) expansion to T4 staging, nodal evaluation, and post-treatment assessment, (4) investigation of CT-AI as a triage or multi-modal prediction tool, and (5) clinical impact and cost-effectiveness analyses to demonstrate real-world value and inform implementation strategies. Regarding model interpretability, the current framework relies on the U-Net generated segmentation masks to visualize the tumor location. This explicit delineation provides radiologists with direct evidence of the region being analyzed. Future iterations may incorporate additional attention visualization techniques, such as Grad-CAM, specifically for the staging network. Finally, to address the challenge of variability across different CT scanners and protocols, future work will explore advanced domain adaptation and dynamic learning strategies. Recent methodologies in intelligent control and unsupervised adaptation, such as those proposed for dynamic network environments [23] and prototype learning [24], offer conceptual frameworks that could be adapted to improve the robustness of medical AI systems in complex, real-world clinical settings.

Finally, the translation of deep learning models from research to routine clinical practice requires careful assessment of algorithmic safety and robustness. Recent studies have highlighted that medical AI systems in cross-sectional imaging—including both MRI and CT—may be vulnerable to specific reliability issues. These include hallucination-like failure modes, in which models produce confident but incorrect predictions, and sensitivity to adversarial perturbations, where subtle input modifications may lead to erroneous outputs [25,26,27]. Although our study employed a supervised learning framework with radiologist-verified ground truth to improve label reliability, safe clinical deployment will still require robustness evaluation, uncertainty estimation, and human-in-the-loop verification strategies to ensure patient safety in real-world settings.

## 5. Conclusions

This study demonstrates that a CT-based AI system can feasibly detect, segment, and stage rectal cancer, with staging accuracy comparable to radiologists. These findings support its feasibility as a clinical decision-support tool and highlight the need for further validation and workflow integration. Further multi-center and prospective validation is warranted.

## Figures and Tables

**Table 1:** Comparison of baseline characteristics between the training/validation cohort and the test cohort.

Variable	Training + Validation (*n* = 177)	Test (*n* = 46)	*p*-Value
Age (years, mean ± SD)	62.8 ± 11.5	63.7 ± 9.4	0.65
Sex, male (%)	104 (59%)	30 (65%)	0.72
Tumor size (cm, mean ± SD)	4.01 ± 1.3	3.80 ± 1.1	0.27
CEA (ng/mL, median [IQR])	2.71 [1.51–5.34]	2.67 [1.61–5.92]	0.59
CT–surgery interval (days, mean ± SD)	6.6 ± 9.8	3.5 ± 4.4	0.002 *

* *p* < 0.05, statistically significant.

**Table 2:** Performance of AI models in rectal cancer detection, segmentation, and staging.

Task	Model	Metric	Result
Lesion detection	RCD-CNN	Accuracy	97.6%
		Recall	97.5%
		Precision	97.6%
Segmentation	U-Net (rectal contour)	Dice score	0.897
	U-Net (tumor localization)	Dice score	0.856
Staging	RCS-3DCNN	Accuracy	80.4% (95% CI: 66.8–89.3)
		Sensitivity	73.7%
		Specificity	85.2%

RCD-CNN: rectal cancer detection convolutional neural network; RCS-3DCNN: rectal cancer staging 3D convolutional neural network; U-Net: U-shaped convolutional neural network architecture; Dice: Dice similarity coefficient; CI: confidence interval.

**Table 3:** Comparison of AI and Radiologist Staging Performance Against Pathology.

Metric	Radiologist	AI (RCS-3DCNN)	*p*-Value ^a^
Accuracy	82.6% (38/46)	80.4% (37/46)	1.0
Sensitivity (T3)	94.7% (18/19)	73.7% (14/19)	—
Specificity (T12)	74.1% (20/27)	85.2% (23/27)	—
Cohen’s κ	0.66	0.59	—
AI–Radiologist agreement	76.1% (35/46)	—	—
Accuracy when agreed	94.3% (33/35)	—	—

ᵃ McNemar’s exact test (two-sided).—indicates not applicable. AI, artificial intelligence; RCS-3DCNN, Rectal Cancer Staging Three-Dimensional Convolutional Neural Network; κ, Cohen’s kappa coefficient; T12, pathological stage T1 or T2; T3, pathological stage T3.

## Data Availability

The data presented in this study are available on request from the corresponding author. The de-identified CT images and corresponding annotations used in this study are not publicly available due to institutional privacy policies and ethical restrictions. Data may, however, be made available to qualified researchers upon reasonable request to the corresponding author, subject to approval by the Institutional Review Board and completion of a formal data transfer agreement. The model architecture specifications and evaluation code are available from the corresponding author upon request to facilitate reproducibility.

## Abbreviations

The following abbreviations are used in this manuscript:

AI	Artificial Intelligence
CNN	Convolutional Neural Network
RCD-CNN	Rectal Cancer Detection Convolutional Neural Network
RCS-3DCNN	Rectal Cancer Staging Three-Dimensional Convolutional Neural Network
CT	Computed Tomography
ROI	Region of Interest
IRB	Institutional Review Board
HU	Hounsfield Unit
MRI	Magnetic Resonance Imaging
SD	Standard Deviation
CI	Confidence Interval
T12	Pathological stage T1 or T2
κ	Cohen’s kappa coefficient
CEA	Carcinoembryonic antigen
IQR	Interquartile Range
